# MicroRNA-29a Alleviates Bile Duct Ligation Exacerbation of Hepatic Fibrosis in Mice through Epigenetic Control of Methyltransferases

**DOI:** 10.3390/ijms18010192

**Published:** 2017-01-18

**Authors:** Ya-Ling Yang, Feng-Sheng Wang, Sung-Chou Li, Mao-Meng Tiao, Ying-Hsien Huang

**Affiliations:** 1 Department of Anesthesiology, Kaohsiung Chang Gung Memorial Hospital and Chang Gung University College of Medicine, Kaohsiung 83301, Taiwan; yaling453@yahoo.com.tw; 2 Core Laboratory of Phenomics & Diagnostics, Department of Medical Research, Kaohsiung Chang Gung Memorial Hospital and Chang Gung University College of Medicine, Kaohsiung 83301, Taiwan; wangfs@ms33.hinet.net; 3 Genomics and Proteomics Core Laboratory, Department of Medical Research, Kaohsiung Chang Gung Memorial Hospital and Chang Gung University College of Medicine, Kaohsiung 83301, Taiwan; raymond.pinus@gmail.com; 4 Department of Pediatrics, Kaohsiung Chang Gung Memorial Hospital and Chang Gung University College of Medicine, Kaohsiung 83301, Taiwan; tmm@adm.cgmh.org.tw

**Keywords:** miR-29a, bile duct ligation, cholestasis, liver fibrosis, methyltransferase, genetic methylation, PTEN

## Abstract

MicroRNA-29 (miR-29) is found to modulate hepatic stellate cells’ (HSCs) activation and, thereby, reduces liver fibrosis pathogenesis. Histone methyltransferase regulation of epigenetic reactions reportedly participates in hepatic fibrosis. This study is undertaken to investigate the miR-29a regulation of the methyltransferase signaling and epigenetic program in hepatic fibrosis progression. miR-29a transgenic mice (miR-29aTg mice) and wild-type littermates were subjected to bile duct-ligation (BDL) to develop cholestatic liver fibrosis. Primary HSCs were transfected with a miR-29a mimic and antisense inhibitor. Profibrogenic gene expression, histone methyltransferases and global genetic methylation were probed with real-time quantitative RT-PCR, immunohistochemical stain, Western blot and ELISA. Hepatic tissue in miR-29aTg mice displayed weak fibrotic matrix as evidenced by Sirius Red staining concomitant with low fibrotic matrix collagen 1α1 expression within affected tissues compared to the wild-type mice. miR-29a overexpression reduced the BDL exaggeration of methyltransferases, DNMT1, DNMT3b and SET domain containing 1A (SET1A) expression. It also elevated phosphatase and tensin homolog deleted on chromosome 10 (PTEN) signaling within liver tissue. In vitro, miR-29a mimic transfection lowered collagen 1α1, DNMT1, DNMT3b and SET1A expression in HSCs. Gain of miR-29a signaling resulted in DNA hypomethylation and high PTEN expression. This study shines a new light on miR-29a inhibition of methyltransferase, a protective effect to maintain the DNA hypomethylation state that decreases fibrogenic activities in HSC. These robust analyses also highlight the miR-29a regulation of epigenetic actions to ameliorate excessive fibrosis during cholestatic liver fibrosis development.

## 1. Introduction

Chronic liver injuries caused by hepatitis and cholestasis lead to liver fibrosis, a detrimental reaction regulated by several signaling pathways [1]. Trans-differentiation of hepatic stellate cells (HSCs) is a prominent feature in the development of liver fibrosis. The HSCs, a cell population with vitamin A storage capacity, are activated and shifted toward contractile myofibroblastic cells that produce excessive extracellular matrices (ECM) during hepatic fibrosis [1,2,3,4].

Accumulating evidence has revealed that low expression of the microRNA-29 (miR-29) family is linked to the occurrence of liver fibrosis and cirrhosis in clinical specimens and animal models [4,5,6,7]. HSC cultures in an activated state express high fibrogenic reactions in association with low expression of miR-29a, b and c [5]. miR-29 overexpression attenuates collagen and fibrotic matrix accumulation in HSCs through directly targeting genes of interest [5,8]. We previously uncovered that miR-29a overexpression mitigated hepatocellular apoptosis and HSC activation during liver injury [1,6,7]. miR-29a reduction of TGF-β1 and histone deacetylase 4 signaling diminished profibrogenic phenotypes of HSCs and, thereby, ameliorated bile duct-ligation (BDL)-mediated cholestatic liver fibrosis [1,6].

Epigenetic activities of genomes in various cell types include chromatin structure remodeling, DNA methylation and acetylation and microRNA actions [9]. DNA methylation is found to facilitate or inactivate the modification of lysine residues in promoter regions [10]. Several DNA methyltransferases (DNMTs) are observed to participate in DNA methylation reactions [11]. Of interest, methylation of histone [12] and DNA are linked to the activation of HSCs. Control of epigenetic activity by the DNA methylation inhibitor modulates hepatic wound healing and fibrogenesis [9,10]. In addition, the miR-29 family has been found to target DNMT3A and DNMT3B and curtail the aberrant methylation status of DNA in non-small-cell lung cancer cells [11]. The crosstalk of miR-29a, histone and DNA methyltransferases in cholestatic liver tissue remains elusive. We hypothesized that DNA methyltransferase may be involved in the miR-29a reduction of liver fibrosis. In this study, we used miR-29a transgenic mice (miR-29aTg mice) to test whether miR-29a changed methyltransferase signaling or DNA methylation state during the BDL-mediated hepatic injuries and fibrogenesis.

## 2. Results

### 2.1. Overexpression of miR-29a Alleviated Fibrosis in Cholestatic Livers

**Figure 1:**
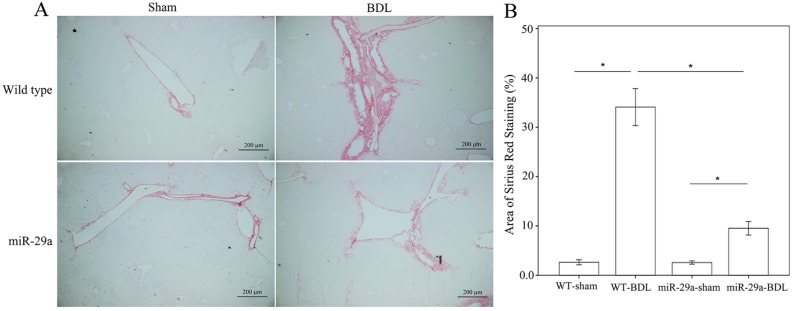
Overexpression of miR-29a resulted in the downregulation of liver fibrosis in mice following bile duct-ligation (BDL). (**A**) Histochemical images of Sirius Red staining in liver tissue. Specimens in the wild-type (WT) mice showed intensive fibrosis after BDL, whereas hepatic tissue in the miR-29aTg mice exhibited mild fibrosis, which was limited to the immediate vicinity of the portal area; (**B**) Data calculated from the six to eight samples per group are expressed as the mean ± SE. * Indicates a *p* < 0.05 between the groups.

**Figure 2:**
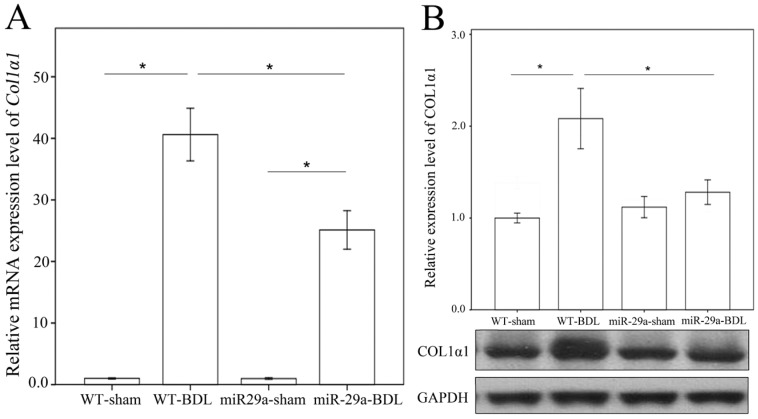
Analyses of collagen 1α1 mRNA (**A**) and protein (**B**) expressions in the WT and miR-29Tg mice livers following BDL and sham operations. Data calculated from six to eight samples per group are expressed as the mean ± SE. * Indicates a *p* < 0.05 between the groups. GAPDH indicates glyceraldehyde 3-phosphate dehydrogenase.

We examined ECM deposition and production with Sirius Red staining to confirm whether miR-29a overexpression changed the sequela of cholestatic liver injuries. As shown in Figure 1, the injured liver around the portal area in the wild-type (WT) mice exhibited evident fibrosis at one week after BDL, whereas the miR-29aTg mice showed minor fibrotic matrix formation. Analyses of qRT-PCR and Western blot revealed significant increases in mRNA and protein expression of collagen1α1 in the BDL-WT group compared with those in the sham group (*p* < 0.001; Figure 2A,B). In the miR-29aTg mice, the BDL-mediated collage 1α1 mRNA expression and protein levels were significantly reduced (*p* < 0.001, respectively).

### 2.2. miR-29a Overexpression Reduced DNA Methyltransferases, Histone Methyltransferase and SET Domain Containing 1A in Cholestatic Mice

**Figure 3:**
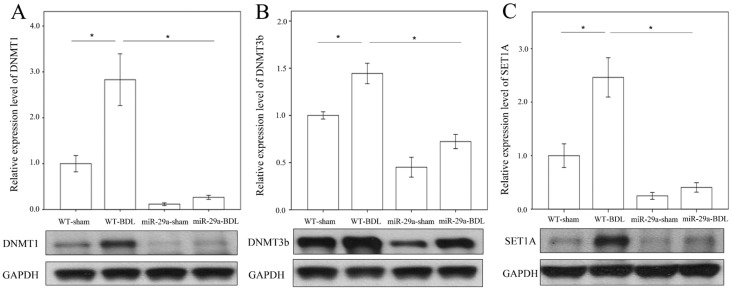
Analyses of DNMT1 (**A**), DNMT3b (**B**) and SET1A (**C**) abundances in affected livers in the WT and miR-29Tg mice following BDL. Data calculated from the six to eight samples per group are expressed as the mean ± SE. * Indicates a *p* < 0.05 between the groups.

We further examined whether BDL changed the concentrations of DNA methyltransferase or histone methyltransferase protein in the hepatic tissue. As revealed in Figure 3, the BDL-WT group exhibited an increase in DNMT1, DNMT3b and SET1A protein levels compared to those in the sham operation group (*p* < 0.001, *p* = 0.002, *p* < 0.001, respectively). In the BDL-miR-29aTg group, the abundances of DNMT1, DNMT3b and SET1A were significantly lower than those in the BDL-WT group (*p* < 0.001, respectively), which was suggestive of the active responses of these molecules to miR-29a signaling in early cholestasis.

### 2.3. miR-29a Overexpression Increased PTEN and Lowered PI3K Signaling in Cholestatic Livers

**Figure 4:**
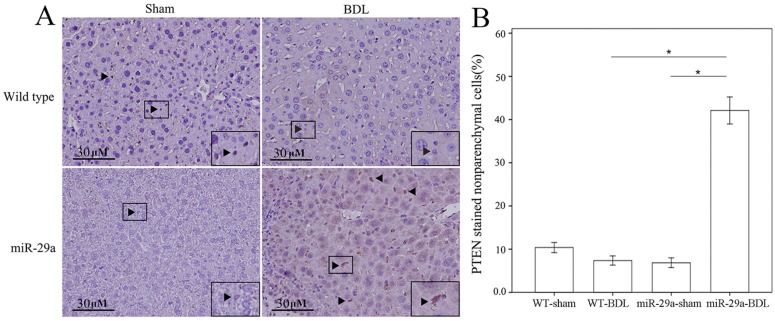
Histomorphometric analyses of phosphatase and tensin homolog deleted on chromosome 10 (PTEN) immunostaining in cholestatic livers. (**A**) Specimens in the miR-29aTg mice showed strong PTEN immunoreactivity in nonparenchymal cells (brown color, insert) compared to those in the WT littermates; (**B**) Number of liver cells positive for PTEN immunoreactivity. Data are expressed as the mean ± SE of the six to eight samples per group. * Indicates a *p* < 0.05 between the groups.

**Figure 5:**
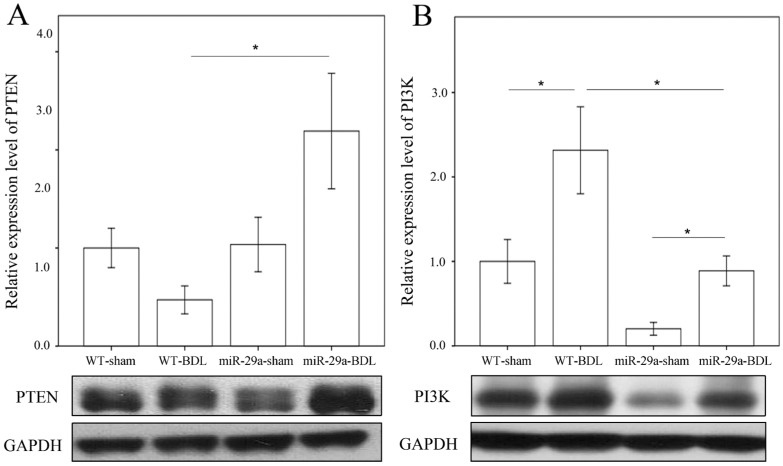
Immunoblotting analyses of tensin homolog deleted on chromosome 10 (PTEN) (**A**) and phosphatidylinositide 3-kinases (PI3K); (**B**) levels in livers of the WT and miR-29Tg mice following BDL. Data calculated for the six to eight samples per group are expressed as the mean ± SE. * Indicates a *p* < 0.05 between the groups.

Phosphatase and tensin homolog deleted on chromosome 10 (PTEN) signaling is found to prevent HSC activation and induce apoptosis [12,13,14]. Targeting PI3K/AKT signaling exhibited a negative impact on HSC proliferation and activation [14]. We tested whether miR-29a attenuation of liver fibrosis was linked to PTEN and PI3K signaling. Immunohistochemical analyses revealed that nonparenchymal liver cells exhibited strong PTEN immunoreactivity (arrowhead) concomitant with a significant elevation in the number of liver cells positive for PTEN immunoactivity in the miR-29aTg group (Figure 4). Consistent with histological investigations, the BDL-miR29Tg group exhibited a significant increase in PTEN protein levels as compared with those in the WT group (Figure 5A, *p* < 0.001).

In the BDL-WT group, liver tissue showed a significant increase in PI3K concentration as compared with the sham-WT group (Figure 5B; *p* = 0.006). A significant reduction in basal PI3K levels was noted in the miR-29Tg mice (*p* = 0.003). The BDL-mediated elevation of PI3K levels was significantly reduced in the miR-29Tg group (*p* < 0.001).

### 2.4. miR-29a Regulated DNA Methylation and Histone Methyltransferases Expressions in Hepatic Stellate Cells

**Figure 6:**
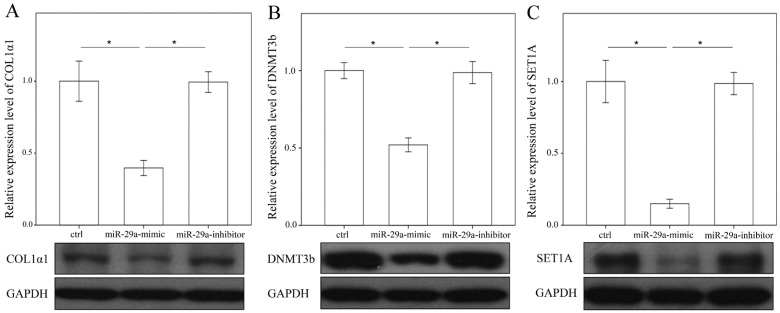
Overexpression of miR-29a decreased COL1A1 (**A**), DNMT3b (**B**) and SET1A (**C**) expressions in primary hepatic stellate cells (HSCs). Data from the six to eight samples per group are expressed as the mean ± SE. * Indicates a *p* < 0.05 between the groups.

**Figure 7:**
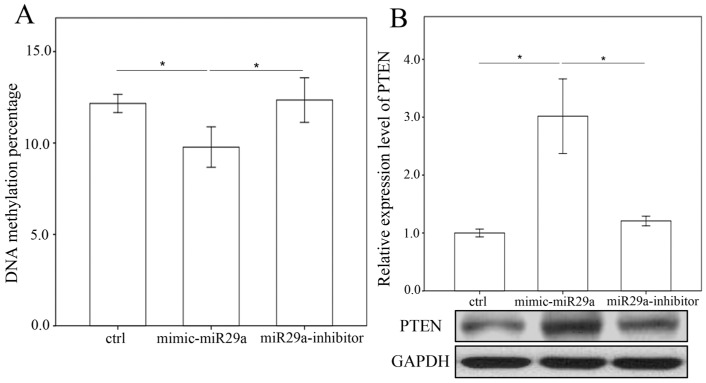
Analyses of DNA methylation (**A**) and tensin homolog deleted on chromosome 10 (PTEN) (**B**) on 10th culture day of activated HSCs after treatment with a miR-29a mimic and anti-sense inhibitor for 24 h. Data are expressed as the mean ± SE of the six to eight samples per group. * Indicates a *p* < 0.05 between the groups.

To determine the effects of miR-29a inhibition on the expression of methyltransferases, we adopted primary HSCs to stably express a miR-29a mimic, antisense inhibitor and scramble control. As hypothesized, the gain of miR-29a considerably lessened the expression of collagen 1α1, DNMT3b and SET1A protein expressions in primary HSCs (Figure 6). However, no DNMT1 protein expressions were found in the primary HSCs (data not shown). Analyses of methylated the DNA colorimetric quantification assay revealed that miR-29a overexpression significantly reduced global DNA methylation (Figure 7A). Promotor hypermethylation contributes to the inactivation of PTEN [14]; likewise, the current results showed that gain of miR-29a signaling significantly upregulated PTEN protein expressions in primary HSCs (Figure 7B; *p* < 0.001).

## 3. Discussion

**Figure 8:**
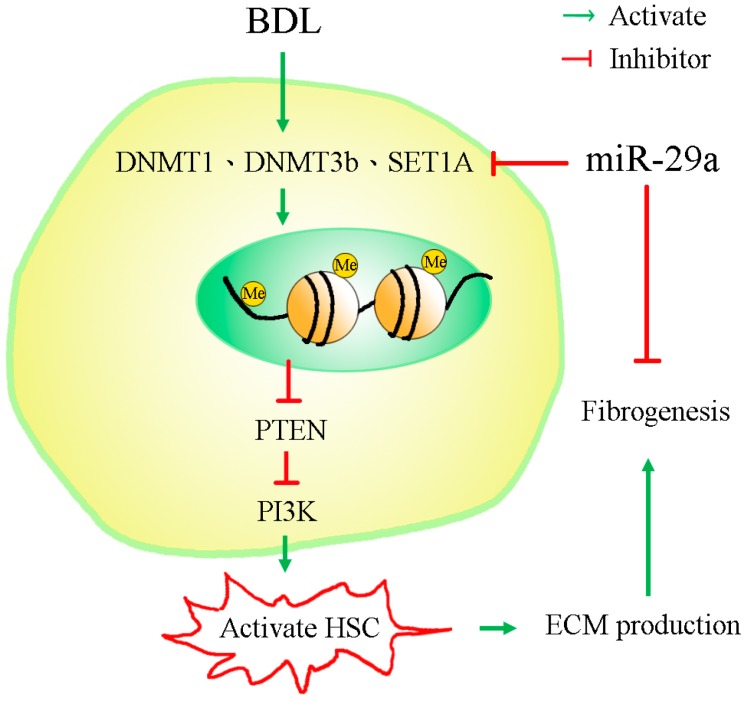
The proposed model of miR-29a signaling protection in liver fibrosis through epigenetic control of methyltransferases. miR-29a is an important regulator of the profibrogenic phenotype of hepatic stellate cells (HSCs). By suppressing methyltransferases action, miR-29a increases PTEN and suppresses phosphatidylinositide 3-kinases (PI3K), thus inhibiting the activation of HSCs. BDL indicates bile duct ligation and ECM indicates extracellular matrices.

Trans-differentiation and activation of HSCs contributes to ECM production, a prominent feature in the pathogenesis of liver fibrosis. Gene methylation and methyltransferases have also been shown to play important roles in HSCs activation [15,16]. As far as we know, this study is the first indication that miR-29a affected DNA methylation and histone methyltransferases signaling in a mouse model of obstructive jaundice. It also highlights the miR-29a actions as follows: (1) an increase in miR-29a in cholestatic mice significantly hindered liver fibrosis; (2) miR-29a overexpression resulted in significant reductions in DNMT1, DNMT3b and SET1A protein expressions in affected livers [17]; (3) a significant elevation in PTEN and reduced PI3K were observed in HSCs in the miR-29aTg mice with cholestatic liver injuries; (4) overexpression of miR-29a caused HSCs to exhibit low expression of collagen 1α1, DNMT3b and SET1A and a high abundance of PTEN; (5) analyses revealed that miR-29a overexpression resulted in the hypomethylation status of DNA in HSCs (Figure 8).

Epigenetic lesions cause changes in both the chromatin structure and the DNA methylation and acetylation patterns of a genome [18]. Thorough knowledge of the involvement of non-coding RNAs in epigenetic mechanisms underlying HSC activation may facilitate the understanding of the pathogenesis of liver fibrosis [19]. In a recent study, Sheen-Chen et al. demonstrated that the biological role of epigenetic methylation of chromatin histone in the BDL induced TGF-β1 expression. Pharmacologic regulation of histone methylation elicits a protective effect against the BDL-induced cirrhosis [9]. DNA methylation is linked to the shift of HSCs into hepatic myofibroblasts. Treatment with DNA methylation inhibitors modulates epigenetic reactions, which mitigates hepatic wound healing and fibrogenesis [9,10]. The miR-29a family controls DNMT3a and DNMT3b gene expression through post-transcriptional pathways [20]. In addition, HNF-4α regulates miR-29a and b signaling that results in low DNMT3 and DNMT3b levels and thereby maintains hepatocyte identity. Bioinformatics searches (http://microrna.sanger.ac.uk and www.microrna.org) revealed that DNMT3A and DNMT3B are putative targets of miR-29a.

Moreover, Galli et al. have shown that the polyinosinic:polycytidylic acid-mediated activation of TLR3 enhances the miR-29 actions to DNA methyltransferases, which results in the demethylation and re-expression of the oncogenesis-suppressor retinoic acid receptor β (RARβ) [21]. HSCs in healthy livers store 80% of total liver retinols. Release of retinols by HSCs depends on extracellular retinol status in hepatic microenvironments. Enhancement of RAR signaling is found to prevent from HSCs activation [22]. Furthermore, methyl CpG binding protein 2 (MeCP2)-dependent epigenetic pathways are observed to hinder HSCs transdifferentiation into the myofibroblast lineage through DNA methyltransferase modulation of peroxisome proliferator-activated receptor gamma gene methylation [23]. PTEN signaling has been reported to weaken the activation of HSCs [12,13,14]. Treatment with the DNA methylation inhibitor, 5-aza-2′-deoxycytidine, promotes PTEN gene expression and reduces the hypermethylation of PTEN gene promoter in activated HSCs [14]. Curcumin treatment has been shown to mitigate HSC proliferation and accelerate the apoptotic program by elevating PTEN-dependent DNA hypomethylation [24]. Moreover, miR-29b participated in the curcumin regulation of PTEN gene demethylation [24]. In this study, the gain of miR-29a expression resulted in the downregulation of DNMT3b in cholestatic livers and HSCs in conjunction with robust epigenetic reaction in terms of DNA demethylation, upregulation of PTEN and downregulation of PI3K expression, which protected against HSCs activation. While direct and indirect demethylation of the PTEN promoter may contribute to the miR-29a induction of global DNA hypomethylation in HSCs, the interaction between miR-29a and PTEN in preventing HSC activation during cholestatic liver injury warrants further characterization in the future.

## 4. Materials and Methods

### 4.1. Ethics Statement

The Institutional Animal Care and Use Committee (IACUC) of Chang Gung Memorial Hospital (#2014121009, 10 March 2016) reviewed and approved all animal use protocols. Male C57BL/6 mice (body weight 25–35 g) were obtained from BioLASCO Taiwan Co., Ltd. (Taipei, Taiwan). All animals were housed in an animal facility at 22 °C, with a relative humidity of 55%, in a 12 h light/12 h dark cycle, with both food and sterile tap water ad libitum.

### 4.2. Construction and Breeding of the miR-29a Transgenic Mouse Colony

Transgenic mice that overexpressed miR-29a driven by the phosphoglycerate kinase 1 promoter were bred and housed in a specific pathogen-free rodent barrier, as previously described [1]. The genotype of the transgenic mice was probed with PCR and primers (forward: 5′-GAGGATCCCCTCAAGGATACCAAGGGATGAAT-3′ and reverse 5′-CTTCTAGAAGGAGTGTTTCTAGGTATCCGTCA-3′). Wild-type mice were obtained from littermates that did not bear the construct.

### 4.3. Animal Model and Experimental Protocol

Six to eight mice were used for all of our experiments. The mice were categorized into either the “BDL” group or the “sham” group, based on whether the mice had received a ligation or a sham ligation of the common bile duct, the method of which has been described in a previous study [7]. All of the mice were euthanized at 1 week postoperatively. Liver tissues were dissected, snap-frozen and processed to isolate total RNA and proteins. All specimens were stored at −80 °C until the biochemical analysis.

### 4.4. Primary HSC Isolation and Culture

Primary HSCs were isolated from fresh livers in mice. In brief, hepatic specimens were digested by pronase and collagenase. The digest mixtures were subjected to density gradient centrifugation in 8.5% Nycodenz (Sigma-Aldrich, St. Louis, MO, USA) as previously described [25,26]. HSCs expressed autofluorescence of retinoids in lipid droplets of cell cultures. HSC lipid droplets were verified under a fluorescence microscope. Trypan blue exclusion assays demonstrated that the viability of cell culture was more than 95%. 95% to 99% of cells were positive for Oil red O staining [26]. Cells were incubated in Dulbecco’s Modified Eagle’s Medium supplemented with 5% newborn calf serum. After one day in culture, the HSCs exhibited a dormant phenotype, followed by an activated phenotype at 7–14 days after incubating. Cell cultures were incubated till confluence; and those within 2–6 passages were used for the study. Each cell culture experiment was carried out at least 6 times.

### 4.5. RNAi Transfection

HSCs were seeded into 6-cm dishes (9 × 10^5^ cells/dish) overnight and then transfected with a miR-29a precursor (a miR-29a mimic, GE Healthcare Dharmacon, Inc., Lafayette, CO, USA), miR-29a antisense oligonucleotide (GE Healthcare Dharmacon, Inc.) or miR control (GE Healthcare Dharmacon, Inc.) for 24 h using the Lipofectamine™ RNAiMAX Transfection Reagent (Invitrogen, Carlsbad, CA, USA), in accordance with the manufacturer’s instructions [27].

### 4.6. RNA Isolation and Real-Time Quantitative RT-PCR

The liver tissue was snap-frozen in liquid nitrogen and ground into a powder for extraction of total RNA using TRIzol reagent (Invitrogen). Two micrograms of total RNA were used for reverse transcription-PCR synthesis with oligo dT primer according to the manufacturer’s instructions. Real-time PCR amplification of reverse-transcription mixtures was performed using LightCycler 480 (Roche Diagnostics, Mannheim, Germany), LightCycler 480 SYBGREEN (Roche Diagnostics) and initial amplification with a denaturation step was set at 95 °C for 5 min, followed by 40 cycles of denaturation at 95 °C for 1 min, annealing at 60 °C for 30 s, extension at 72 °C for 45s and final extension at 72 °C for 5 min. The comparative threshold cycle (*C*_t_) method and 2^−(Δ*C*t target–Δ*C*t calibrator)^ or 2^−ΔΔ*C*t^ were used to expression the relative quantification of gene expression. Primers were designed to amplify collagen-1α1 (forward, 5′-ACCCTGGAAACAGACGA-3′; reverse, 5′-TTTGGTAAGGTTGAATGCACT-3′) and GAPDH (forward, 5′-CACTGCCACCCAGAAGA-3′; reverse, 5′-TCCACGACGGACACATT-3′). We performed validation experiments in duplicate and also validated the amplification efficiencies.

### 4.7. Immunohistological Analysis

For immunohistochemical analysis, formalin-fixed paraffin-embedded blocks of the mice’s liver tissue were cut into 2-µm sections. After deparaffinization and rehydration, sections were heated in a citrate buffer (10 mM, pH 6, Thermo Fisher Scientific, Waltham, MA, USA) in a microwave for 30 min to retrieve the antigens. Endogenous peroxidase activity was blocked with a 3% hydrogen peroxide (UltraVision Hydrogen Peroxide Block; Thermo Fisher Scientific) for 10 min. The sections were then incubated with a PTEN antibody (Santa Cruz, Santa Cruz, CA, USA) for 1 h at room temperature. PTEN immunoreactivity in sections was visualized using HRP polymer (UltraVision Quanto Detection System; Thermo Fisher Scientific) and DAB chromogen (DAB Peroxidase Substrate Kit; Vector Laboratories, Burlingame, CA, USA). The sections were counterstained with Mayer’s hematoxylin (ScyTek Laboratories, Logan, UT, USA), dehydrated and then mounted using a mounting medium.

### 4.8. Western Blot Analysis

The 30-µg protein extracts were mixed with a sample buffer, boiled for 10 min and followed by electrophoresis using 8%–15% sodium dodecyl sulfate-polyacrylamide gels. The proteins in the gels were transferred to a polyvinylidene difluoride membrane. Blots were incubated with primary antibodies against collagen-1α1 (Santa Cruz), DNMT1 (Santa Cruz), DNMT3b (Santa Cruz), SET1A (Santa Cruz), PTEN (Santa Cruz), PI3K (PROTEINTECH, Rosemont, IL, USA) and GAPDH (Santa Cruz) for protein control. After washing the blots with tris-buffered saline and incubating them with horseradish peroxidase-coupled anti-rabbit immunoglobulin-G antibodies (dilution, 1:5000), HRP anti-mouse immunoglobulin-G antibodies (dilution, 1:10,000) and HRP anti-goat immunoglobulin-G antibodies (dilution, 1:10,000) at room temperature for 1 h, we developed them with enhanced chemiluminescence detection (GE Healthcare Biosciences AB, Uppsala, Sweden), exposed them to film and quantified the signals using densitometry.

### 4.9. Genetic Methylation Study

For global DNA methylation, we applied a methylated DNA Colorimetric Quantification Kit (Abcam, Cambridge, UK) pursuant to the manufacturer’s instructions by using 300 ng of DNA per reaction.

### 4.10. Statistical Analysis

All values in the figures and tables are expressed as the mean ± standard error. Quantitative data were analyzed using the one-way analysis of variance [28] as appropriate, while we adopted the least significant difference (LSD) test for post-hoc testing. A two-sided *p*-value less than 0.05 was considered statistically significant.

## 5. Conclusions

Reducing the profibrogenic effects of HSCs is an emerging strategy for prevention from the progression of hepatic fibrosis [29,30]. MicroRNA-mediated epigenetic reactions are unique mechanisms [19,31]. Analytic results of the current study highlight that the miR-29a precursor will be an innovative therapeutic potential for liver fibrosis in the future.
